# Identifying Sources of Faecal Contamination in a Small Urban Stream Catchment: A Multiparametric Approach

**DOI:** 10.3389/fmicb.2021.661954

**Published:** 2021-06-29

**Authors:** Liam J. Reynolds, Niamh A. Martin, Laura Sala-Comorera, Kevin Callanan, Padraig Doyle, Clare O’Leary, Paul Buggy, Tristan M. Nolan, Gregory M. P. O’Hare, John J. O’Sullivan, Wim G. Meijer

**Affiliations:** ^1^UCD School of Biomolecular and Biomedical Science, UCD Earth Institute, and UCD Conway Institute, University College Dublin, Dublin, Ireland; ^2^Central Laboratory, Dublin City Council, Dublin, Ireland; ^3^Drainage Planning, Policy and Development Control, Dublin City Council, Dublin, Ireland; ^4^Municipal Services, Dún Laoghaire-Rathdown County Council, Dublin, Ireland; ^5^UCD School of Computer Science, UCD Earth Institute, University College Dublin, Dublin, Ireland; ^6^UCD School of Civil Engineering, UCD Dooge Centre for Water Resources Research, UCD Earth Institute, University College Dublin, Dublin, Ireland

**Keywords:** faecal contamination, multiparametric, microbial source tracking, urban stream, ammonia, total oxidised nitrogen, phosphorous, sentinel sampling

## Abstract

Small urban streams discharging in the proximity of bathing waters may significantly contribute to the deterioration of water quality, yet their impact may be overlooked. This study focuses on the Elm Park stream in the city of Dublin that is subject to faecal contamination by unidentified sources. The aim of the study was to identify a minimum number of “sentinel” sampling stations in an urban catchment that would provide the maximum amount of information regarding faecal pollution in the catchment. Thus, high-resolution sampling within the catchment was carried out over the course of 1 year at 11 stations. Faecal indicator bacteria were enumerated and microbial source tracking (MST) was employed to evaluate human pollution. In addition, ammonium, total oxidised nitrogen, and phosphorus levels were monitored to determine if these correlated with faecal indicator and the HF183 MST marker. In addition, the effect of severe weather events on water quality was assessed using automated sampling at one of the identified “sentinel” stations during baseflow and high flow conditions over a 24-h period. Our results show that this urban stream is at times highly contaminated by point source faecal pollution and that human faecal pollution is pervasive in the catchment. Correlations between ammonium concentrations and faecal indicator bacteria (FIB) as well as the human MST marker were observed during the study. Cluster analysis identified four “sentinel” stations that provide sufficient information on faecal pollution in the stream, thus reducing the geographical complexity of the catchment. Furthermore, ammonium levels strongly correlated with FIB and the human HF183 MST marker under high flow conditions at key “sentinel” stations. This work demonstrates the effectiveness of pairing MST, faecal indicators, and ammonium monitoring to identify “sentinel” stations that could be more rapidly assessed using real-time ammonium readouts to assess remediation efforts.

## Introduction

Faecal pollution in the environment, including rivers and streams, is a growing problem globally. This is in part due to an increase in urbanisation: 55% of the world’s population currently live in urban areas and this is projected to increase to 68% by 2050 (UN, 2019). Numerous studies have demonstrated that rivers and streams flowing through urban areas are impacted to a greater extent by faecal pollution than their upstream catchments (Goto and Yan, 2011; Paule-Mercado et al., 2016). As faecal pollution may introduce various pathogens, the degradation of such streams and rivers and the bathing waters they discharge into represents an increased infection risk for individuals (Arnold et al., 2017; Kauppinen et al., 2017). For example, a survey of 654 surfers in San Diego revealed increased numbers of wound and gastroenteritis infections following seawater exposure, particularly after rainfall (Arnold et al., 2017). Furthermore, faecal contamination of rivers and streams may negatively impact water used for purposes other than recreational activities, for example irrigation and drinking water intake.

Understanding how faecal pollution enters urban rivers and streams is vital if remediation measures are to be effectively implemented by water management authorities (Walker et al., 2015; Tillett et al., 2018). However, monitoring and remediating faecal pollution is difficult as pollutants can enter via multiple point and diffuse sources (Muller et al., 2020). For example, stream banks, impervious surface cover, and streambed sediments may act as reservoirs of faecal bacteria (Brinkmeyer et al., 2015; Baral et al., 2018; Fluke et al., 2019). Combined sewage overflows (CSOs) and misconnected sanitation pipes also contribute to faecal contamination particularly in cities reliant on antiquated subterranean sewage infrastructure (Brinkmeyer et al., 2015; Balleste et al., 2020). As such, devising efficient and effective sampling and monitoring programmes is vital if authorities are to successfully improve water quality in rivers and streams. A limited number of studies have attempted catchment level surveys to identify high impacted sites in catchments (Sauer et al., 2011; Tillett et al., 2018). Tillett et al., for instance coupled the monitoring of *Escherichia coli*, ammonium and the BacHum (human marker) to identify multiple point sources of human faecal pollution in the Frankston and Mornington Peninsula, Australia (Tillett et al., 2018). However, such sampling campaigns are labour-, time-, and cost-intensive and so water management authorities typically monitor only the discharge points of rivers and streams which provides no data on how and where faecal pollution enters.

Faecal pollution in water bodies is commonly determined by quantifying levels of fecal indicator bacteria (FIB), namely *E. coli* and intestinal enterococci (Saxena et al., 2015). Indeed, the EU bathing water directive uses only these indicators to determine bathing water quality (EU, 2006; Fewtrell and Kay, 2015). As part of the water framework directive (WFD), which aims to improve the ecological and chemical quality of European waters, ammonium, oxidised nitrogen compounds, and phosphorus concentrations are monitored in rivers and streams (EC, 2000). Several studies have identified strong correlations between ammonium, its oxidised derivatives (nitrites and nitrates) and phosphates with FIB (Cabral and Marques, 2006; Mallin and McIver, 2012; Napier et al., 2017; Baral et al., 2018). Although nutrient markers may have non-faecal origins, they offer utility as cost-effective faecal indicators in well characterised catchments where their association with faecal pollution has been validated. Further, monitoring of nutrients can be accomplished by installing probes at relevant stations in a catchment which allows for more rapid identification of faecal pollution events than FIB enumeration. Monitoring only FIB and nutrients, however, provides no information on the biological source of pollution which may be human or zoonotic in origin. Microbial source tracking (MST) methods rely on faecal bacteria being more closely associated with one animal host than another to identify the biological sources of faecal pollution (Harwood et al., 2014). Thus, coupling MST with quantitative PCR (qPCR) can identify the most abundant of these bacteria to provide insight into the primary biological sources of faeces in an environment (Gourmelon et al., 2007; Unno et al., 2018).

The complexity involved in identifying the sources of faecal pollution highlights the need to design and implement effective and efficient monitoring programmes of rivers and streams. Thus, the aim of this study was to use hierarchical clustering to identify a minimum number of “sentinel” stations that provide maximum information about the pollution status of a complex urban stream. Furthermore, as monitoring FIB and MST markers are time consuming, correlation analyses were conducted to determine if specific nutrients could be used as indicators for human faecal pollution events. This study therefore describes methodology to identify a limited number of “sentinel” stations in a complex catchment and an approach to validate ammonium as a marker for faecal contamination within the catchment. This would allow the assessment of water quality in real-time.

## Materials and Methods

### Study Area and Sampling

Dublin bay is a UNESCO Biosphere as it is home to rare wildlife species and is unique in that it encompasses an area of a capital city: Dublin, Ireland. There are also a number of bathing areas within Dublin bay which are popular with tourists and the city’s 560,000 population. Several small streams with completely urban catchments flow through the city and discharge into the bay. The 3.8 km long Elm Park stream, for example, flows through an urban area with a population of approximately 40,000 people before discharging onto Sandymount Strand, a designated bathing water, and the recently declassified Merrion Strand.

**FIGURE 1:**
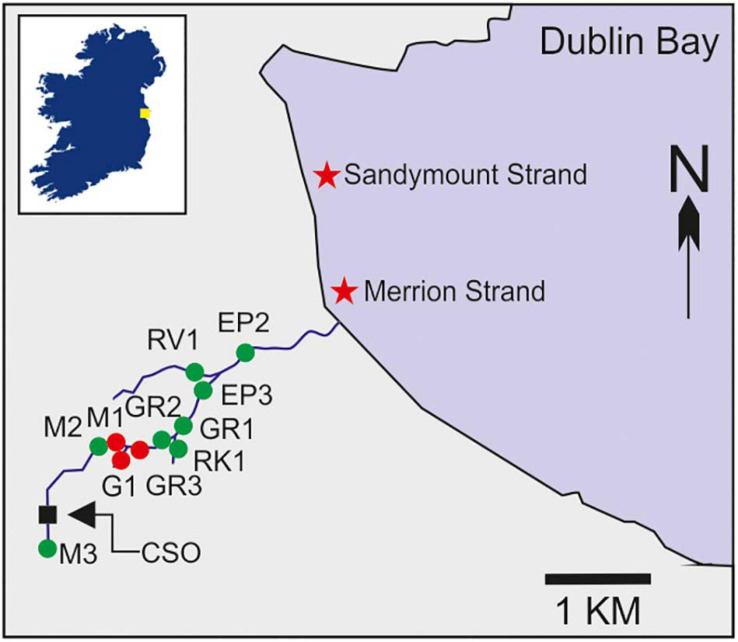
Map of the Elm Park stream catchment indicating the 11 stations sampled as part of the high-resolution survey of the catchment (labelled green and red) as well as the Larchfield combined sewer overflow (CSO). Coordinates of all stations are listed in Supplementary Table 1. The inset box shows the location of Dublin Bay in Ireland indicated by a yellow square. Stations M1, G1, and GR3, which were sampled intensively during stormflow and baseflow, are highlighted in red. Two nearby bathing waters are indicated by stars.

Between October 2019 and September 2020, water samples (*n* = 209) were collected from 11 stations along the Elm Park stream catchment (Figure 1). Faecal pollution in this stream is regularly monitored at its discharge point only. Our sampling regime represented a more detailed assessment of the catchment which we refer to as high-resolution sampling throughout the text. Of these stations, M1–M3 (upstream), together with GR1–3 and EP2–3 (downstream) were located on the main trunk of the stream. Stations G1, RK1, and RV1 were tributaries entering the main trunk (Supplementary Table 1). Duplicate grab samples were collected in sterile 1 L bottles, stored at 4°C, and processed within 6 h.

Automatic samplers with refrigeration units were launched at three upstream stations on the Elm Park Stream (M1, G1, and GR3) during stormflow conditions in February 2020 and baseflow conditions in June 2020 (Figure 1). Baseflow conditions do not significantly vary (*P* = 0.1–0.6) throughout the year. These stations are immediately downstream of a CSO and frequent sampling was conducted to determine how faecal indicators responded to stormflow (CSO active) and baseflow (CSO inactive). During stormflow 23 and 22 samples were collected at M1 and G1, respectively. Due to a machine error 9 samples were collected at GR3, but the peak of stormflow was still captured. During baseflow 24 samples were collected at M1 and G1 and 23 samples were collected at GR3. During these events 900 ml of water was collected hourly from each station in individual sterile bottles and kept at 4°C. Samples were received and processed within 18 h. Modelled rainfall data was obtained from the Met Éireann Re-Analysis database.

### Enumeration of Faecal Indicator Bacteria

Faecal indicator bacteria were enumerated for samples M1–M3, G1, and GR3 by the Dublin City Council Central Laboratory. *E. coli* were enumerated for these samples using the IDEXX Colilert-18 assay according to ISO 9308-2:2012. Briefly, 10 and 100 ml of sample were mixed with dehydrated Colilert medium and incubated in Quanti-Trays at 36.0 ± 2.0°C. *E. coli* concentrations for these samples are determined as most probable number per 100 ml (MPN/100 ml). *E. coli* concentrations for samples GR1–2, EP2–3, RK1, and RV1 were determined using standard filtration methods in University College Dublin according to ISO 16649-1:2018. Samples were passed through 0.45 μm nitrocellulose filter membranes (Nalgene, Thermo Scientific) and then incubated on Tryptone Bile X-Glucuronide agar (TBX; Sigma-Aldrich) for 4 h at 37°C followed by incubation for 18 h at 44°C to enumerate *E. coli* as colony forming units per 100 ml (CFU/100 ml). Previously, split sampling of stations in the Elm Park stream catchment and subsequent analysis by both laboratories demonstrated these protocols to be comparable, therefore *E. coli* concentrations are referred to as CFU/100 ml throughout (Supplementary Figure 1). For all samples, intestinal enterococci were enumerated by incubating filter membranes on Slanetz and Bartley agar (Oxoid) at 37°C for 44 h. Filter membranes were then transferred to Bile Aesculin Agar (Sigma-Aldrich) and incubated at 44°C for 2 h to enumerate intestinal enterococci as CFU/100 ml according to ISO 7899-2:2000. For sites M1–M3, G1, and GR3 the upper limit of quantification was 20,000 MPN/100 ml.

### Extraction of DNA and MST Quantification

Water samples (100 ml) were concentrated by filtration through 0.22 μm nitrocellulose filter membranes and stored at −20°C in cold lysis buffer (5 M guanidine isothiocyanate, 100 mM EDTA [pH 8.0], 0.5% [w/v] sodium lauroyl sarcosinate). A previously described modified DNeasy Blood and Tissue kit protocol (Qiagen) was used to extract DNA from filter membranes (Gourmelon et al., 2007).

Previous work has demonstrated that the HF183 marker is sensitive and specific to human faecal pollution (Boehm et al., 2013; Balleste et al., 2020). The HF183 MST marker was amplified by PCR and cloned into a pBLUE plasmid. This plasmid was linearized and used to create standard curves between 10^6^ and 10^0^ gene copies in each qPCR run to quantify target gene levels in each sample (Supplementary Table 2; Seurinck et al., 2005). Ten minute preincubations at 95°C and melting curve analyses were included in all qPCR cycles. The efficiency of each reaction was determined using the *E* = 10^(1/slope)^ − 1 equation (Rutledge and Côté, 2003). The limit of detection was determined as the lowest concentration of DNA detected in 95% or more of replicates and the limit of quantification was determined as the lowest concentration of DNA quantified within 0.5 SDs of the log_10_ concentration (Supplementary Table 2; Rutledge and Stewart, 2008). All standard curves had an *R*^2^ value greater than 0.985.

Immediately following DNA extraction, duplicate qPCR reactions from undiluted and 10-fold diluted samples for each DNA extraction were conducted. All 20 μl reaction mixtures contained 10 μl of SYBR Green I Master (Roche), 500 nM each of the forward and reverse primer (Supplementary Table 2), 1 μl of sample, and were run on the Roche Lightcycler 96 platform (Roche). HF183 MST marker concentrations were expressed as gene copies per 100 ml (gc/100 ml).

### Nitrogen Compound and Phosphorus Analysis

All nitrogen and phosphorus compounds were analysed using Gallery Plus instrumentation with an automated discrete colorimetric analyser. Phosphorus pollution was determined by measuring orthophosphate levels using the automated ascorbic acid reduction method with absorbance measured at 880 nm. Orthophosphate concentrations were derived as mg/L (as P) and the quantification limit for this assay is 0.01 mg/L (as P) (Baird and Bridgewater, 2017).

All nitrogen compound concentrations were derived as mg/L (as N). Ammonium concentration in all samples was determined using the automated phenate method with absorbance being monitored at 660 nm, the quantification limit for this assay is 0.01 mg/L (as N) (Baird and Bridgewater, 2017). Nitrite concentrations were determined using the automated sulphanilamide – *N*-1-Naphthylethylene-diamine dihydrochloride (NEDD) colorimetric method, with absorbance measured at 540 nm, the quantification limit for this assay is 0.005 mg/L (as N) (Baird and Bridgewater, 2017). Total oxidized nitrogen (TON) was monitored by first reducing the nitrate in the sample to nitrite. The produced nitrite and the nitrite present in the sample was then quantified using the automated sulphanilamide – NEDD colorimetric method and the quantification limit for this assay is 0.01 mg/L (as N). The nitrate concentrations of samples were derived by subtracting the nitrite concentration from the TON concentration (Baird and Bridgewater, 2017).

### Multivariate and Statistical Analyses

Hierarchical clustering is a method that groups objects such that intra-cluster objects are more similar than between cluster objects. Principle component analysis is a process of data reduction that can identify the main variables contributing to variation of a dataset. By coupling hierarchical clustering and principal component analysis, “sentinel” stations and the primary variables contributing to them can be identified. Hierarchical clustering and principal component analysis were accomplished using scaled and log transformed data in RStudio 3.5.1. Hierarchical clustering of stations based on the geometric means of the assessed variables was conducted using Ward’s hierarchical linkage of Euclidean distances (Suzuki and Shimodaira, 2006). Multiscale bootstrapping was employed to determine “true” clusters (>0.90 significance cut-off). Principal component analysis was conducted with Horn’s Parallel Analysis to determine the minimum number of significant principal components. Varimax rotation was employed on the resulting principal components to identify the primary variables contributing to each principal component.

Spearman correlation analyses of the levels of FIB, MST markers, and nutrient concentrations were performed using Prism GraphPad software (version 9.1.0.221). A significance cut-off of *p* ≤ 0.05 was used for all analyses. When culture, molecular, and chemical assays had an upper limit of quantification this upper limit was used in statistical analyses when reached. When culture, molecular, and chemical assays where below the quantification limit, a value of half the quantification limit was used in further analyses.

## Results and Discussion

### High-Resolution Sampling Identifies Hotspots of Human Faecal Pollution and Informs the Identification of “Sentinel” Stations

**FIGURE 2:**
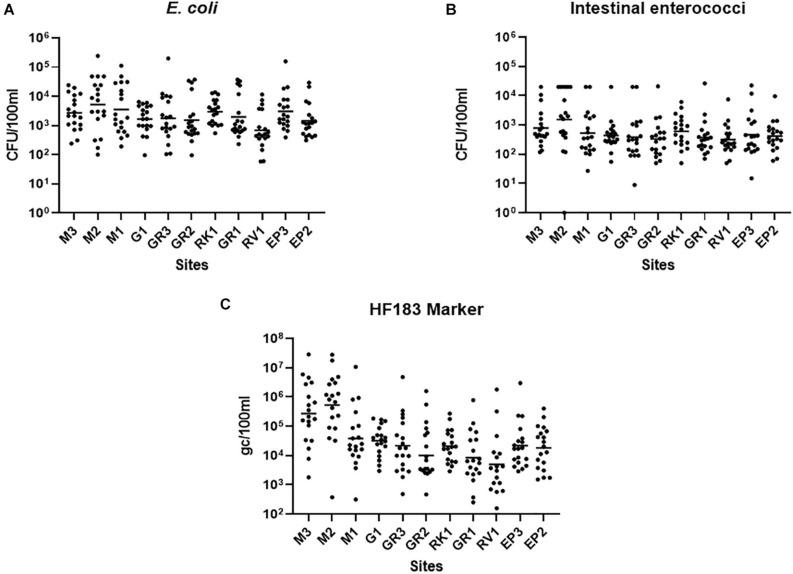
Faecal Indicator bacteria concentrations in the Elm Park Catchment. Shown are the levels of panels **(A)** *E. coli*, **(B)** intestinal enterococci, and **(C)** the HF183 (human) microbial source tracking marker at sampling station. The dot plots show the concentration of each sample and the geometric means are indicated as a horizontal line.

Faecal pollution in urban streams and rivers can have a detrimental impact on the bathing waters into which they discharge (Molina et al., 2014). For remediation measures to be successful in protecting bathing waters understanding faecal pollution and devising efficient monitoring programmes are paramount (Walker et al., 2015). Thus, high-resolution sampling of the Elm Park stream was conducted to better understand faecal pollution in the catchment and to inform the identification of “sentinel” stations. All sampling stations in the Elm Park stream were highly polluted as FIB concentrations varied up to four orders of magnitude (5.90 × 10^1^–2.42 × 10^5^ and <10–2.65 × 10^4^ CFU/100 ml for *E. coli* and intestinal enterococci, respectively) (Figures 2A,B). On occasion, these levels surpassed those observed in treated wastewater impacted rivers and streams that have been studied (Paule-Mercado et al., 2016; Balleste et al., 2020). High-resolution sampling of the Elm Park catchment revealed station M2, which is located directly downstream of a CSO, to have the highest concentrations of *E. coli* (geometric mean of 5.16 × 10^3^ CFU/100 ml) and intestinal enterococci (geometric mean of 1.54 × 10^3^ CFU/100 ml). Although the concentrations of FIB generally decreased along the course after this point of the stream, the geometric mean of concentrations of *E. coli* and intestinal enterococci never fell below 1.4 × 10^3^ and 3 × 10^2^ CFU/100 ml, respectively, in the main trunk of the stream. These data suggest that there are no significant point sources of pollution between station M2 and GR3. However, as the levels of FIB remained high between these stations, they are likely impacted by upstream flow and surface run-off from the embankment (Muller et al., 2020). Interestingly, *E. coli* concentrations increased again at station EP3 (geometric mean of 3,054 CFU/100 ml). As there are no documented CSOs between GR1 and EP3, this increase indicates the presence of an unknown point source of faecal pollution. The tributary stations, G1 and RV1, typically had FIB concentrations lower than, or at comparable levels to downstream stations on the main trunk. The RK1 tributary station on the other hand had a higher mean concentration of both faecal indicators than GR1 which is immediately downstream and may be contributing to faecal pollution in the main trunk.

In addition to identifying the geographical sources of faecal pollution, it is important to determine the primary biological sources of faecal pollution, as human pollution poses a greater risk to human health than animal faecal pollution (Soller et al., 2010). The human faecal marker, HF183, could be quantified in all but two samples (RV1 samples collected in August 2020), demonstrating that human faecal pollution is pervasive in the Elm Park catchment (Figure 2C). HF183 concentrations along the Elm Park stream followed a similar pattern to that of the FIB, although, the variation observed was greater, up to 10^6^-fold at some stations. Station M3 and M2 exhibited the highest HF183 concentrations (geometric means of 5.28 × 10^5^ and 2.74 × 10^5^ gc/100 ml, respectively) providing further evidence of upstream pollution in the Elm Park catchment and that M2 is directly impacted by the CSO discharging human faecal waste. Station M3 is not impacted by any documented point sources so human faecal pollution is likely entering the stream from diffuse leakages and misconnections. On occasion, these two upstream stations had HF183 concentrations approaching that documented for raw sewage (10^8^ gc/100 ml) (Hughes et al., 2017; Balleste et al., 2020). Although HF183 concentrations decreased along the main trunk of the Elm Park stream by greater than 10-fold at M1 (3.81 × 10^4^ gc/100 ml) and greater than 60-fold at GR1 (8.00 × 10^3^ gc/100 ml), these levels are still considered high and within the range of other human waste impacted streams (Olds et al., 2018).

In this study, high-resolution sampling was coupled with MST analysis in the Elm Park catchment to identify point and diffuse sources of human faecal pollution at stations M3, M2, and EP3. Such an approach may prove useful to identify locations of faecal pollution in other urban stream or larger river catchments (Tillett et al., 2018).

### High-Resolution Sampling Reveals Catchment Level Nitrogen and Phosphorus Contamination

**FIGURE 3:**
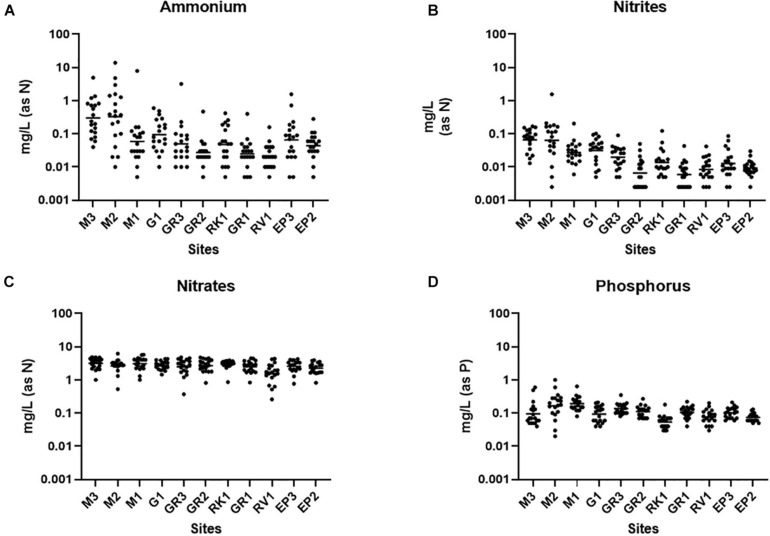
Nutrient concentrations in the Elm Park Catchment. Shown are the levels of panels **(A)** ammonium, **(B)** nitrite, **(C)** nitrate, and **(D)** phosphorus for each sampling station. The dot plots show the concentration of each sample.

Ammonium is a parameter that is monitored as part of the WFD and has also been used to identify incidences of faecal pollution (EC, 2000; Cabral and Marques, 2006; Mallin and McIver, 2012). Nutrient markers such as ammonium, when used alongside FIB and MST, can assist in identifying the biological source of faecal pollution and may offer an alternative and more rapid method of monitoring faecal pollution (Lim et al., 2017). Ammonium concentrations varied up to four orders of magnitude at some stations in the Elm Park catchment (Figure 3A). The highest concentrations of ammonium were observed at stations M3 and M2 [0.01–13.91 mg/L (as N); geometric mean of 0.3298 mg/L (as N)] which are comparable to treated wastewater impacted rivers (Jin et al., 2017; Preisner, 2020). This agrees with the high concentrations of FIB and HF183 observed at these upstream stations suggesting that ammonium levels may be indicative of human faecal pollution in this catchment. Moving downstream, concentrations of ammonium generally decreased in the Elm Park catchment such that all downstream stations had geometric means ≤ 0.1 mg/L (as N). Nitrite in surface waters can result from faecal pollution and from the oxidation of ammonium by ammonium oxidising bacteria and archaea (He et al., 2018). Nitrite levels in the Elm Park catchment followed the same trend as ammonium with concentrations ranging over three orders of magnitude [>0.001 to >1 mg/L (as N)] (Figure 3B). Thus, increased nitrite concentrations may be indicative of faecal pollution in the Elm Park catchment. In contrast, little variation was observed in nitrate levels within the catchment [geometric mean of 1.539–3.133 mg/L (as N)] which typically made up greater than 96% of measured TON (Figure 3C). Nitrates are present in human excreta and are the terminal oxidation product of ammonium. They may also enter surface water from decaying plant matter and atmospheric deposition (Vrzel et al., 2016). The temporal and geographical stability of nitrate concentrations in the Elm Park catchment indicates that it has little use as a faecal indicator. Phosphorus is also present in human excreta, but it can also enter surface water by mineral erosion and organic decomposition (van der Kooij et al., 2020). Phosphorus concentrations exhibited little variation and so may not prove useful as a faecal indicator in the Elm Park catchment [geometric means of 0.05–0.19 mg/L (as P)] (Figure 3D).

### Faecal Indicator Bacteria, HF183, and Ammonium Strongly Correlate at Highly Polluted Stations

If alternative nutrient parameters are to be successfully used as rapid alternatives to monitor faecal pollution it is important to determine their relationship with classical FIB and MST markers. We therefore carried out correlation analyses. At station M3, which is highly impacted by human faecal pollution, no correlations between FIB, MST markers, and nutrients were observed. Faecal pollution at this station originates from diffuse human sources which may be sporadic in nature. Thus, variations in pollutant discharge coupled with different die-off rates between FIB and MST harbouring *Bacteroides* spp. will compound correlations between these indicators and nutrient parameters. Similarly, no correlations were observed at tributary stations G1 and RK1.

**FIGURE 4:**
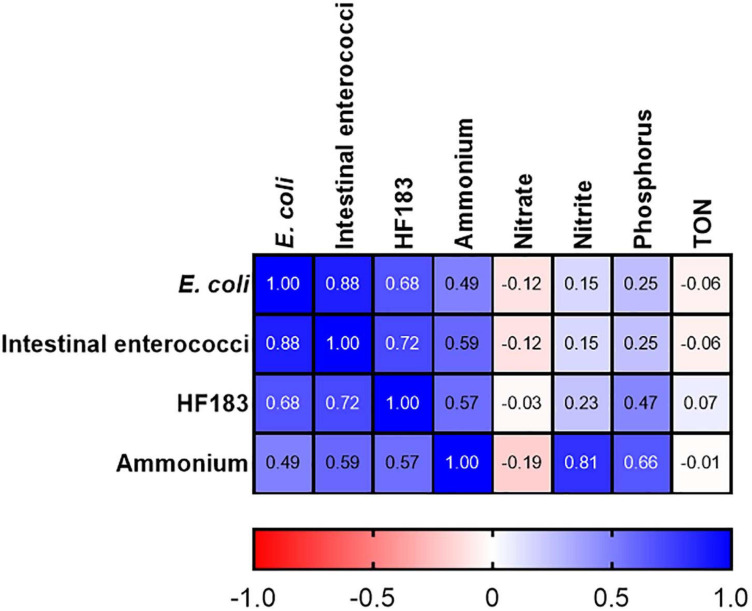
Heatmap showing correlations between FIB, the HF183 marker and nutrients at station M2. TON refers to total oxidised nitrogen. Dark blue and dark red indicate strong positive and negative correlations, respectively. *R*^2^ coefficients > 0.49 were considered statistically significant (*p* < 0.05).

At station M2 statistically significant correlations between FIB, the human MST marker, and ammonium were observed (*R*^2^ = 0.491–0.879; *p*-value <0.05) (Figure 4). Previous studies have demonstrated a strong correlation between ammonium, *E. coli*, intestinal enterococci, and HF183 in pooled WWTP influent/effluent (Mayer et al., 2016). As M2 is directly impacted on by a CSO, the strong correlations documented between ammonium concentrations and FIB as well as HF183 demonstrates the usefulness of this nutrient as a proxy for recent faecal pollution that can be measured in a rapid and cost-effective manner. In well characterised catchments, where ammonium has been shown to correlate with faecal indicators, this would allow for real-time monitoring of remediation efforts that would complement regular monitoring of FIB and MST markers. Downstream of station M2, on the main trunk of the stream, *E. coli* and intestinal enterococci concentrations correlated (*R*^2^ = 0.507–0.800; *p*-value <0.05), however, correlations with HF183 and ammonium were more sporadic (Supplementary Table 3). Indeed, no correlations with these indicators are observed at the most downstream EP3 and EP2 stations. As these stations are furthest geographically from the M2 CSO this may be a result of dilution of the CSOs effluent.

At stations GR2 and GR3 FIB levels correlated with ammonium but only with HF183 concentrations at M1 (*R*^2^ = 0.537–0.703; *p*-value <0.05). At the tributary stations, only correlations between intestinal enterococci and *E. coli* concentrations were observed at RV1 (*R*^2^ = 0.606; *p*-value <0.05). No statistically significant correlations were observed between nitrate, TON and Phosphorus, and the faecal indicators at any stations. As these nutrients exhibited little variation this is expected and further supports these parameters being unsuitable as faecal indicators in the Elm Park catchment. These results indicate the usefulness of monitoring ammonium to determine faecal pollution in the catchment, particularly, following recent pollution events.

### Cluster Analysis of High-Resolution Sampling Data Identifies “Sentinel” Stations

**FIGURE 5:**
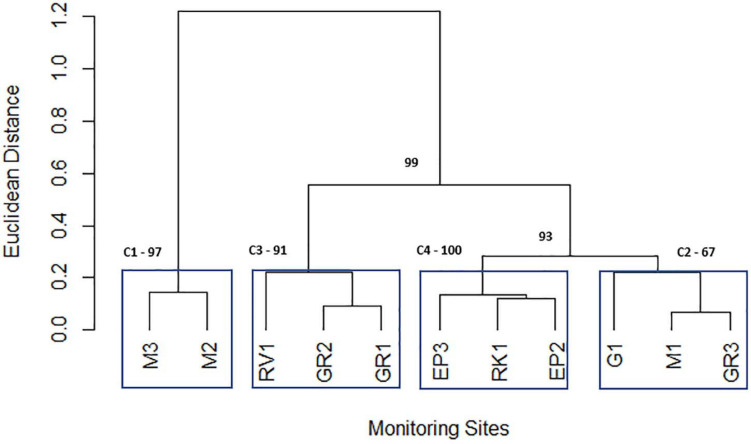
Dendrogram depicting the grouping of Elm Park catchment sampling stations obtained from the geometric means of variables using the Ward hierarchical clustering of Euclidean distances. Clusters are labelled C1, C2, C3, and C4 and surrounded by blue boxes. The numbers highlighted in bold indicate the *p*-values (approximately unbiased *p*-value; cut-off > 90%) used to identify “true” clusters.

Cluster analysis of the geometric mean of each variable for each station resulted in the 11 stations being grouped into 4 statistically significant clusters. These four clusters represent the geographical grouping of the sampling stations of the Elm Park catchment. Cluster 1 (M3 and M2) represents the most upstream stations in the catchment, both cluster 2 (M1, G1, and GR3) and cluster 3 (GR2, GR1, and RV1) are in the middle of the catchment and cluster 4 (EP3, EP2, and RK1) typifies the culverted and downstream stations of the stream (Figure 5). Sampling of representative “sentinel” stations from each cluster may allow more efficient and rapid monitoring of faecal pollution in the Elm Park stream going forward as just four stations would be sampled rather than all 11. Nnane et al., employed a similar approach on the River Ouse in the United Kingdom whereby they reduced the 14 stations to 5 or 6 “sentinel” stations (Nnane, 2011; Nnane et al., 2011). Furthermore, as the tributaries (G1, RK1, and RV1) didn’t group into distinct clusters this further supports the observations that faecal pollution is occurring predominantly in the main trunk of the Elm Park stream.

Principal component analysis was performed on individual clusters to identify the main influential variables of each (Figure 5). Three principal components were retained for each cluster according to Horn’s Parallel analysis. Interestingly, for all four clusters, PC2 represented between 19 and 26% of the total variation and was driven by moderate loadings (>0.65) of nitrates and phosphorus (Supplementary Table 4). These variables may represent embankment erosion indicating that all clusters are impacted by diffuse runoff to comparable extents (Mellander et al., 2018). PC1 of cluster 1 (which represented 37% of variance) was primarily accounted for by positive moderate ammonium, nitrite, and TON loadings (>0.56). The positive loading of ammonium in this principal component indicates that it represents domestic sewage pollution in cluster 1 (Cabral and Marques, 2006; Baral et al., 2018). Indeed, M2 is downstream of a CSO and M3 is likely impacted by diffuse domestic faecal pollution. PC1 represents 37 and 60% of the variation of cluster 2 and 3, respectively. In both cases moderate negative (−0.638 to −0.537) loadings of *E. coli* and intestinal enterococci are the primary factors. This indicates that stream volume, that dilutes these FIB, is a key factor for these clusters, particularly cluster 3. PC1 of cluster 4 (explaining 32% of variation) has moderate negative loadings (−0.600 to −0.583) of ammonium and nitrite. This cluster includes the stations EP3 and EP2 that are furthest from the CSO (which is key contributor of ammonium to the catchment) suggesting FIB influx at these stations is not concomitant with ammonium pollution.

This approach of using hierarchical clustering of high-resolution sampling data to identify “sentinel” stations can provide water management authorities with a novel and more efficient faecal pollution monitoring system in other catchments.

### Stormflow Influences Faecal Indicator Pollution and Correlates With Ammonium Concentrations in the Upstream Elm Park Catchment

**FIGURE 6:**
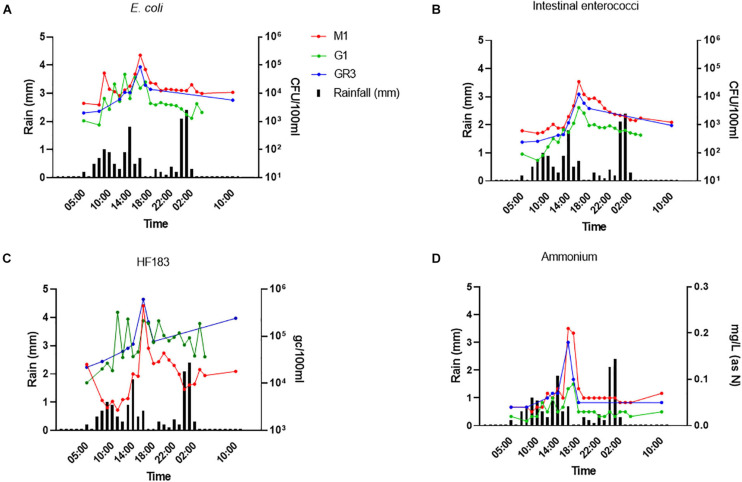
The response of *E. coli* **(A)**, intestinal enterococci **(B)**, HF183 **(C)**, and **(D)** ammonium concentrations to rainfall at stations GR3, G1, and M1. Modelled rainfall is depicted as mm of rain per hour.

**FIGURE 7:**
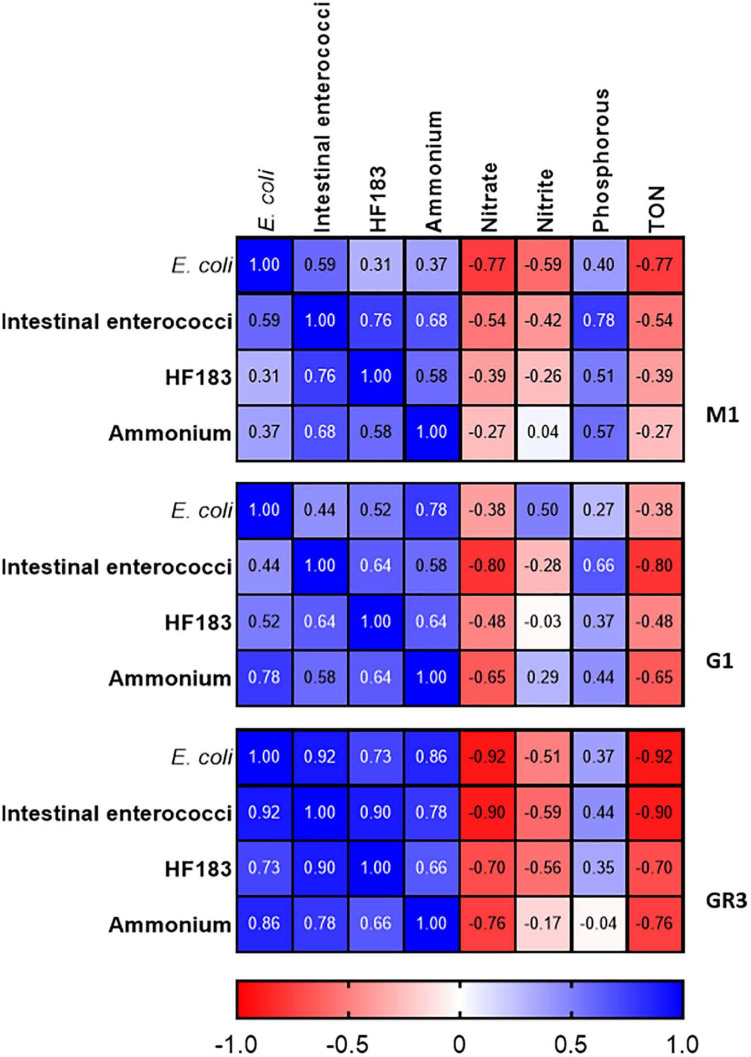
Heatmap showing correlations between FIB, the HF183 marker and nutrients at station M1, G1, and GR3 during stormflow. Dark blue and dark red indicate strong positive and negative correlations, respectively. *R*^2^ coefficients > 0.49 were considered statistically significant (*p* < 0.05).

To further assess the utility of ammonium and other nutrient parameters as rapid alternatives to FIB and MST marker monitoring, intensive sampling under stormflow (February 2020) and baseflow (June 2020) conditions at the three cluster 2 stations (M1, G1, and GR3) was undertaken. Over the stormflow period, three peaks in rainfall were modelled, whereas no rainfall was modelled during baseflow. During stormflow conditions, M1 and the downstream GR3 station responded similarly to increased rainfall. Peak concentrations of FIB in response to rainfall were observed at hour 10 of the storm at both stations, for *E. coli* (4.70 × 10^4^–2.25 × 10^5^ CFU/100 ml), intestinal enterococci (4.10 × 10^3^–3.47 × 10^4^ CFU/100 ml), HF183 (3.24 × 10^5^–6.10 × 10^5^ gc/100 ml), and ammonium [0.09–0.21 mg/L (as N)] (Figures 6A–D). Faecal indicator concentrations increased between 10- and 100-fold whereas ammonium concentrations increased over threefold. At station G1 intestinal enterococci and ammonium concentrations followed a similar response to rainfall as observed at M1 and GR3. Although *E. coli* and HF183 concentrations increased in response to rainfall, their levels were more variable at this station. Such intra-storm variability has been described previously for faecal indicators and antibiotic resistance genes in urban streams (Liao et al., 2015; Garner et al., 2017). Statistically significant correlations (*R*^2^ = 0.437–0.917; *p* < 0.05) between FIB, HF183, and ammonium concentrations were observed at all stations during stormflow (Figure 7 and Supplementary Table 5). Conversely, nitrate, nitrite, and TON followed the opposite trend to ammonium at all three stations in response to rainfall as concentrations decreased until hour 10 of the storm before increasing again. In fact, statistically significant negative correlations between nitrate and TON and FIB and the HF183 marker were observed (*R*^2^ = −0.917 to −0.538; *p* < 0.05). Together, these data suggest that increased stream flow during stormflow conditions rapidly introduces ammonium rich human faeces into the Elm Park catchment which concomitantly dilutes oxidised nitrogen compounds. Thus, monitoring ammonium in real-time can be used to identify faecal contamination following storm events which can subsequently be confirmed by standard FIB enumeration.

**FIGURE 8:**
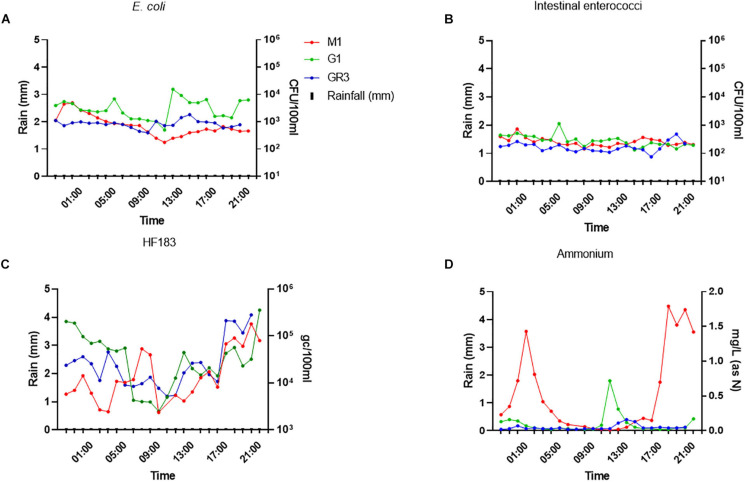
Variation of panel **(A)** *E. coli* **(B)** intestinal enterococci, **(C)** the HF183 marker, and **(D)** ammonium concentrations during baseflow at stations GR3, G1, and M1. Modelled rainfall is depicted as mm of rain per hour.

*Escherichia coli* and intestinal enterococci concentrations at all three stations were more stable during baseflow than stormflow, ranging between 10^2^–10^4^ and 10^2^–10^3^ CFU/100 ml, respectively (Figures 8A,B). In contrast, HF183 concentrations at all stations were more variable and had a diurnal pattern as they decreased until 9–10 a.m. before increasing again until the evening. A similar pattern of diurnality was observed for ammonium concentrations but only at station M1 (Figures 8C,D).

Surprisingly, ammonium concentrations exceeded 1.4 mg/L (as N) twice during baseflow sampling at M1, over sevenfold greater than the peak observed during stormflow conditions. In fact, nitrite and phosphorus concentrations were up to 10-fold higher at all stations during baseflow, which likely results from dilution of these nutrients during rainfall. At station M1, *E. coli* and intestinal enterococci correlated (*R*^2^ = 0.578–0.704; *p* < 0.0038) with nitrate and nitrite concentrations during baseflow, which contrasts with stormflow observations (Supplementary Table 5). This station is closest to the M2 CSO and so these correlations indicate that the CSO may be slowly discharging sewage into the stream during baseflow. Under such conditions the sewage associated ammonium will be oxidised to nitrate and nitrite in the environment.

Together the monitoring of cluster 2 sampling stations during stormflow and baseflow revealed that all stations exhibit similar patterns of variability in faecal indicator levels, particularly in response to rainfall. This provides further evidence that sampling a single “sentinel” station from a cluster provides maximum information for that cluster. Furthermore, the strong correlations observed between FIB, MST, and ammonium levels highlight the effectiveness of monitoring ammonium as a rapid alternative to FIB and MST.

## Conclusion

To conclude, we have demonstrated the usefulness of conducting high-resolution sampling to obtain a detailed picture of faecal pollution in a catchment. Further to this, we have demonstrated how these data can be used to reduce the geographical complexity of a catchment by employing hierarchical clustering to identify “sentinel” sampling stations. This “sentinel” sampling approach coupled with real-time monitoring of nutrients that correlate with faecal pollution in the catchment, such as ammonium, provides an alternative, and efficient approach to water management authorities to assess remediation efforts.

## Data Availability Statement

The raw data supporting the conclusion of this article will be made available by the authors, without undue reservation.

## Author Contributions

LR wrote the draft of the manuscript. LR, NM, LS-C, TN, KC, PD, JO’S, CO’L, and PB carried out the experiments. LR, WM, PD, PB, CO’L, KC, JO’S, and LS-C conceived and planned the experiments. WM received funding for the project and supervised the project. All authors contributed to manuscript revision, read, and approved the submitted version.

## Conflict of Interest

The authors declare that the research was conducted in the absence of any commercial or financial relationships that could be construed as a potential conflict of interest.
