# Supplementary material for: Identifying Sources of Faecal Contamination in a Small Urban Stream Catchment: A Multiparametric Approach
Source: Front Microbiol. 2021 Jun 29;12:661954. doi: 10.3389/fmicb.2021.661954 (PMC8276237; doi:10.3389/fmicb.2021.661954)
Supplement: Supplementary file 1 [file Table_1.docx]

Supplementary Material

**Identifying Sources of Faecal Contamination in a Small Urban Stream Catchment: A Multiparametric Approach**

Liam J. Reynolds^1^, Niamh A. Martin^1^, Laura Sala-Comorera^1^, Kevin Callanan^2^, Padraig Doyle^3^, Clare O’Leary^2^, Paul Buggy^4^, Tristan M. Nolan^1^, Gregory M. P. O’Hare^5^, John J. O’Sullivan^6^, Wim G. Meijer^1*^.

1. UCD School of Biomolecular and Biomedical Science, UCD Earth Institute, and UCD Conway Institute, University College Dublin, Ireland
2. Central Laboratory, Dublin City Council, Eblana House, Marrowbone Lane, Dublin 8, Ireland
3. Drainage Planning , Policy and Development Control, Dublin City Council, Civic Offices, Wood Quay, Dublin 8, Ireland
4. Municipal Services, Dún Laoghaire-Rathdown County Council, County Hall, Marine Road, Dún Laoghaire, Ireland
5. UCD School of Computer Science and UCD Earth Institute, University College Dublin, Belfield, Dublin 4, Ireland
6. UCD School of Civil Engineering, UCD Dooge Centre for Water Resources Research and UCD Earth Institute, University College Dublin, Dublin 4, Ireland.

Key words: Faecal contamination, Multiparametric, Microbial source tracking, Urban stream, Ammonium, Total oxidised nitrogen, phosphorous, sentinel sampling

*Corresponding author.

Tel: (+353) 17162778

Email: wim.meijer@ucd.ie

| Sampling Site | Designation | GPS Location (Latitude, Longitude) |
| --- | --- | --- |
| M3 | Main Trunk (Upstream) | -6.240136, 53.295535 |
| M2 | Main Trunk (Upstream) | -6.235377, 53.304612 |
| M1 | Main Trunk (Upstream) | -6.232842, 53.304932 |
| GR3 | Main Trunk | -6.229976, 53.304497 |
| GR2 | Main Trunk | -6.224438, 53.305643 |
| GR1 | Main Trunk | -6.224324, 53.305741 |
| EP3 | Main Trunk (Downstream) | -6.220279, 53.309904 |
| EP2 | Main Trunk (Downstream) | -6.217258, 53.311149 |
| G1 | Tributary | -6.232361, 53.303766 |
| RK1 | Tributary | -6.224353, 53.305654 |
| RV1 | Tributary | -6.220576, 53.310355 |
| CSO | Larchfield  Combined Sewer Overflow | -6.236454, 53.298850 |

**Table S1.** GPS locations and designation of the sampling sites in this study.

| **Target Gene** | **Primer Sequence** | | **Amplicon Size** | **LoD** | **LoQ** | **Accepted Efficiencies** | **Cycling Condition** | **Reference** |
| --- | --- | --- | --- | --- | --- | --- | --- | --- |
| HF183 | | F: ATCATGAGTTCACATGTCCG  R: TACCCCGCCTACTATCTAATG | 82bp | 1.1 gc/μl | 2.2 gc/μl | 1.95 – 2.0 | 45 cycles (95°C - 5s, 60°C - 15s, 72 - 20s) | ([Bernhard and Field, 2000](#_ENREF_1); [Seurinck *et al.*, 2005](#_ENREF_2)) |

**Table S2.** Primers, target genes, cycling conditions, Limit of Detection (LoD) and Quantification (LoQ) used in this study.

Bernhard, A.E. and K.G. Field, 2000. A pcr assay to discriminate human and ruminant feces on the basis of host differences in *bacteroides*-*prevotella* genes encoding 16s rrna. Applied and environmental microbiology, 66(10): 4571. Available from <http://aem.asm.org/content/66/10/4571.abstract>. DOI 10.1128/AEM.66.10.4571-4574.2000.

Seurinck, S., T. Defoirdt, W. Verstraete and S.D. Siciliano, 2005. Detection and quantification of the human-specific hf183 bacteroides 16s rrna genetic marker with real-time pcr for assessment of human faecal pollution in freshwater. Environmental microbiology, 7(2): 249-259. DOI 10.1111/j.1462-2920.2004.00702.x.

| **Site** | **Faecal Indicator, MST Marker, Ammonium** | ***E. coli*** | **Intestinal enterococci** | **HF183** | **Ammonium** | **Nitrate** | **Nitrite** | **Phosphorus** | **TON** |
| --- | --- | --- | --- | --- | --- | --- | --- | --- | --- |
| M3 | *E. coli* | **-**  0.378  0.381 | 0.378  **-**  0.341 | 0.381  0.341  **-** | 0.213  -0.261  0.085 | -0.309  -0.237  -0.082 | 0.261  -0.007  -0.065 | 0.063 | -0.309 |
|  | IE |  |  |  |  |  |  | -0.316 | -0.237 |
|  | HF183 |  |  |  |  |  |  | 0.097 | -0.082 |
|  | Ammon. | 0.213 | -0.261 | 0.085 | **-** | **-0.565*** | **0.676**** | **0.501*** | **-0.565*** |
| M2 | *E. coli* | **-**  **0.879*****  **0.675****  **0.491*** | **0.879*****  **-**  **0.717****  **0.589**** | **0.675****  **0.717****  **-**  **0.568*** | **0.491***  **0.589****  **0.568***  **-** | -0.117  -0.119  -0.03  -0.191 | 0.154  0.153  0.233  **0.809***** | 0.252  0.247  0.473  **0.660**** | -0.055  -0.062  0.068  -0.014 |
|  | IE |  |  |  |  |  |  |  |  |
|  | HF183 |  |  |  |  |  |  |  |  |
|  | Ammon. |  |  |  |  |  |  |  |  |
| M1 | *E. coli* | **-**  **0.707****  0.133  0.308 | **0.707****  **-**  0.442  0.322 | 0.133  0.442  **-**  **0.537*** | 0.380  0.322  **0.537***  **-** | -0.012  -0.088  0.261  -0.07 | -0.072  -0.249  0.406  **0.599*** | -0.087  0.038  -0.019  0.304 | 0.012  -0.062  0.275  -0.043 |
|  | IE |  |  |  |  |  |  |  |  |
|  | HF183 |  |  |  |  |  |  |  |  |
|  | Ammon. |  |  |  |  |  |  |  |  |
| GR3 | *E. coli* | **-**  **0.507***  0.298  **0.705**** | **0.507***  **-**  **0.562***  0.348 | 0.298  **0.562***  **-**  0.313 | **0.705****  0.348  0.313  **-** | 0.382  0.039  0.332  0.374 | 0.291  0.093  0.244  **0.686**** | -0.239  0.021  -0.078  0.011 | 0.382  0.039  0.332  0.374 |
|  | IE |  |  |  |  |  |  |  |  |
|  | HF183 |  |  |  |  |  |  |  |  |
|  | Ammon. |  |  |  |  |  |  |  |  |
| GR2 | *E. coli* | **-**  **0.800*****  **0.539***  **0.703**** | **0.800*****  **-**  0.352  **0.675**** | **0.539***  0.352  **-**  0.342 | **0.703****  **0.675****  0.342  **-** | 0.285  0.126  0.145  0.313 | **0.511***  0.402  0.441  **0.679**** | -0.217  -0.192  0.080  -0.224 | 0.294  0.134  0.148  0.319 |
|  | IE |  |  |  |  |  |  |  |  |
|  | HF183 |  |  |  |  |  |  |  |  |
|  | Ammon. |  |  |  |  |  |  |  |  |
| GR1 | *E. coli* | **-**  **0.696*****  0.430 | **0.696****  **-**  0.454 | 0.430  0.454  **-** | 0.257  **0.524***  **0.489*** | 0.322  0.182  -0.175 | **0.501***  0.407  0.298 | -0.242 | 0.337 |
|  | IE |  |  |  |  |  |  | -0.223 | 0.185 |
|  | HF183 |  |  |  |  |  |  | 0.155 | -0.161 |
|  | Ammon. | 0.257 | 0.524 | **0.489*** | - | 0.249 | 0.594 | -0.364 | 0.255 |
| EP3 | *E. coli* | **-**  **0.879*****  0.196 | **0.879****  **-**  0.235 | 0.196  0.235  **-** | 0.068  0.131  0.121 | **0.482***  0.425  -0.014 | 0.042  0.063  0.113 | -0.230 | **0.481*** |
|  | IE |  |  |  |  |  |  | -0.087 | 0.428 |
|  | HF183 |  |  |  |  |  |  | -0.029 | -0.025 |
|  | Ammon. | 0.068 | 0.131 | 0.121 | - | 0.017 | **0.764***** | 0.336 | 0.035 |
| EP2 | *E. coli* | **-**  **0.675*****  0.412 | **0.675****  **-**  0.329 | 0.412  0.329  **-** | -0.051  -0.193  0.062 | **0.465***  0.098  -0.026 | 0.286  0.056  0.331 | -0.170 | **0.465*** |
|  | IE |  |  |  |  |  |  | 0.003 | 0.098 |
|  | HF183 |  |  |  |  |  |  | 0.332 | -0.026 |
|  | Ammon. | -0.051 | -0.193 | 0.062 | - | 0.229 | **0.581*** | 0.008 | 0.035 |
| G1 | *E. coli* | **-**  0.377  0.360 | 0.377  **-**  0.360 | 0.360  0.360  **-** | -0.279  -0.154  -0.360 | 0.188  -0.187  -0.049 | 0.385  0.354  0.282 | -0.337 | 0.210* |
|  | IE |  |  |  |  |  |  | 0.051 | -0.158 |
|  | HF183 |  |  |  |  |  |  | 0.005 | -0.045 |
|  | Ammon. | -0.279 | -0.154 | -0.360 | - | -0.382 | **0.856***** | **0.694**** | -0.363 |
| RK1 | *E. coli* | **-**  0.354  0.057 | 0.354  **-**  -0.053 | 0.057  -0.053  **-** | 0.189  0.001  0.239 | 0.018  0.228  0.184 | 0.458  -0.071  0.416 | 0.276 | 0.018* |
|  | IE |  |  |  |  |  |  | -0.338 | -0.228 |
|  | HF183 |  |  |  |  |  |  | 0.181 | -0.184 |
|  | Ammon. | 0.189 | 0.001 | 0.239 | - | 0.442 | **0.783***** | 0.246 | -0.442 |
| RV1 | *E. coli* | **-**  **0.606****  0.245  0.009 | **0.606****  **-**  0.287  0.111 | 0.245  0.287  **-**  0.139 | 0.009  0.111  0.139  - | -0.293  0.078  -0.042  0.379 | 0.179  **0.587***  0.319  0.349 | -0.005  0.079  0.457  **0.510*** | -0.293 |
|  | IE |  |  |  |  |  |  |  | 0.078 |
|  | HF183 |  |  |  |  |  |  |  | -0.042  0.379 |
|  | Ammon. |  |  |  |  |  |  |  |  |

**Table S3.** Correlations Between FIB, the HF183 MST marker and nutrients. * p ≤ 0.05, ** p ≤ 0.005, *** p ≤ 0.001.

| **Variable** | **Cluster 1 Loadings** | | | **Cluster 2 Loadings** | | | **Cluster 3 Loadings** | | | **Cluster 4 Loadings** | | |
| --- | --- | --- | --- | --- | --- | --- | --- | --- | --- | --- | --- | --- |
|  | PC1 | PC2 | PC3 | PC1 | PC2 | PC3 | PC1 | PC2 | PC3 | PC1 | PC2 | PC3 |
| *E. coli* |  |  | -0.632 | -0.568 |  |  | -0.537 |  |  |  |  | 0.627 |
| IE |  |  | -0.620 | -0.636 |  |  | -0.542 |  |  |  |  | 0.673 |
| HF183 |  |  | -0.459 | -0.468 |  |  |  |  | -0.409 |  |  | 0.384 |
| Ammonium | 0.569 |  |  |  |  | -0.515 |  |  |  | -0.583 |  |  |
| Nitrate |  | 0.698 |  |  | 0.671 |  |  | 0.655 |  |  | 0.661 |  |
| Nitrite | 0.603 |  |  |  |  | -0.640 |  |  |  | -0.600 |  |  |
| Phosphorus |  | 0.697 |  |  | 0.670 |  |  | 0.655 |  |  | 0.661 |  |
| TON | 0.545 |  |  |  |  | -0.546 |  |  | -0.835 | -0.472 |  |  |

**Table S4.** The primary loadings (following varimax rotation) contributing to the first three principle components (PC) of each cluster are displayed. Variables contributing to more than one principle component were considered only for the principle component that had the greatest loading.

| **Site** | **Faecal Indicator, MST Marker, Ammonium** | ***E. coli*** | **Intestinal enterococci** | **HF183** | **Ammonium** | **Nitrate** | **Nitrite** | **Phosphorus** | **TON** |
| --- | --- | --- | --- | --- | --- | --- | --- | --- | --- |
| **Stormflow** |  |  |  |  |  |  |  |  |  |
| M1 | *E. coli* | **-**  **0.588****  0.307 | **0.588***  **-**  **0.761***** | 0.307  **0.761*****  **-** | 0.372  **0.684*****  **0.583**** | **-0.774*****  **-0.538****  -0.389 | **-0.587****  **-0.419***  -0.263 | 0.400 | **-0.774***** |
|  | IE |  |  |  |  |  |  | **0.776***** | **-0.538**** |
|  | HF183 |  |  |  |  |  |  | **0.515*** | -0.389 |
|  | Ammon. | 0.372 | **0.684***** | **0.583**** | **-** | -0.389 | 0.039 | **0.571**** | -0.268 |
| G1 | *E. coli* | **-**  **0.437***  **0.517***  **0.777***** | **0.437***  **-**  **0.641****  **0.580**** | **0.517***  **0.641****  **-**  **0.644**** | **0.777*****  **0.580****  **0.644****  **-** | -0.384  **-0.802*****  **-0.481***  **-0.649**** | **0.498***  -0.281  -0.026  0.291 | 0.271  **0.661****  0.372  **0.437*** | -0.384  **-0.802*****  **-0.481***  **-0.649**** |
|  | IE |  |  |  |  |  |  |  |  |
|  | HF183 |  |  |  |  |  |  |  |  |
|  | Ammon. |  |  |  |  |  |  |  |  |
| GR3 | *E. coli* | **-**  **0.917****  **0.733***  **0.861**** | **0.917****  **-**  **0.900***  **0.785*** | **0.733***  **0.900****  **-**  **0.658*** | **0.861****  **0.785***  **0.658***  **-** | **-0.917****  **-0.900****  **-0.700***  **-0.760*** | -0.509  -0.593  -0.559  0.172 | 0.375  0.443  0.349  -0.039 | **-0.917****  **-0.900****  **-0.700***  **-0.760*** |
|  | IE |  |  |  |  |  |  |  |  |
|  | HF183 |  |  |  |  |  |  |  |  |
|  | Ammon. |  |  |  |  |  |  |  |  |
| **Baseflow** |  |  |  |  |  |  |  |  |  |
| M1 | *E. coli* | **-**  **0.511***  0.091  **0.558*** | **0.511***  **-**  -0.178  0.362 | 0.091  -0.178  **-**  **0.503*** | **0.558***  0.362  **0.503***  **-** | **0.689*****  **0.578****  0.001  **0.528*** | **0.704*****  **0.592****  0.168  **0.816***** | **0.457***  **0.223**  **0.587****  **0.696***** | **0.685*****  **0.590****  0.042  **0.575**** |
|  | IE |  |  |  |  |  |  |  |  |
|  | HF183 |  |  |  |  |  |  |  |  |
|  | Ammon. |  |  |  |  |  |  |  |  |
| G1 | *E. coli* | **-**  0.118  **0.467***  **0.404*** | 0.118  **-**  **0.417***  0.323 | **0.467***  **0.417***  **-**  **0.423*** | **0.404***  0.323  **0.423***  **-** | 0.040  -0.178  **0.401***  0.274 | 0.317  0.138  **0.497***  **0.894***** | 0.071  -0.038  0.213  0.342 | 0.050  -0.155  **0.423***  0.312 |
|  | IE |  |  |  |  |  |  |  |  |
|  | HF183 |  |  |  |  |  |  |  |  |
|  | Ammon. |  |  |  |  |  |  |  |  |
| GR3 | *E. coli* | **-**  -0.066  0.064 | -0.066  **-**  **0.621**** | 0.064  **0.621****  **-** | 0.269  0.376  **0.471*** | 0.043  0.300  0.311 | 0.331  **0.410***  **0.520*** | -0.092 | 0.043 |
|  | IE |  |  |  |  |  |  | 0.392 | 0.300 |
|  | HF183 |  |  |  |  |  |  | 0.337 | 0.311 |
|  | Ammon. | 0.269 | 0.376 | **0.471*** | - | **0.632**** | **0.837***** | **0.673**** | **0.632**** |

**Table S5.** Correlations between FIB, the HF183 MST marker and nutrients at sites M1, G1 and GR3 during stormflow and baseflow. Statisticall significant correlations are highlighted in bold. * p ≤ 0.05, ** p ≤ 0.005, *** p ≤ 0.001.

**Figure S1:** Results of split sampling of sites in the Elm Park Stream Catchment. Samples were analysed using the Colilert® (MPN/100ml) method in the Dublin City Council (DCC) laboratory and using the filtration method with cultivation on TBX agar (CFU/100ml) in the UCD laboratory. Both methods were comparable as a strong correlation (R^2^ = 0.9078) between both was observed.

*** p ≤ 0.001.
